# International comparison of emergency hospital use for infants: data linkage cohort study in Canada and England

**DOI:** 10.1136/bmjqs-2016-006253

**Published:** 2017-06-12

**Authors:** Katie Harron, Ruth Gilbert, David Cromwell, Sam Oddie, Astrid Guttmann, Jan van der Meulen

**Affiliations:** 1 London School of Hygiene and Tropical Medicine, London, UK; 2 Great Ormond Street Institute of Child Health, London, UK; 3 Bradford Teaching Hospitals NHS Foundation Trust, Bradford, UK; 4 Institute of Clinical Evaluative Sciences, Toronto, Ontario, Canada; 5 Department of Health Services Research and Policy, London School of Hygiene and Tropical Medicine, London, UK; 6 Clinical Effectiveness Unit, The Royal College of Surgeons of England, London, UK

**Keywords:** emergency department, health services research, hospital medicine, paediatrics, healthcare quality improvement

## Abstract

**Objectives:**

To compare emergency hospital use for infants in Ontario (Canada) and England.

**Methods:**

We conducted a population-based data linkage study in infants born ≥34 weeks’ gestation between 2010 and 2013 in Ontario (n=253 930) and England (n=1 361 128). Outcomes within 12 months of postnatal discharge were captured in hospital records. The primary outcome was all-cause unplanned admissions. Secondary outcomes included emergency department (ED) visits, any unplanned hospital contact (either ED or admission) and mortality. Multivariable regression was used to evaluate risk factors for infant admission.

**Results:**

The percentage of infants with ≥1 unplanned admission was substantially lower in Ontario (7.9% vs 19.6% in England) while the percentage attending ED but not admitted was higher (39.8% vs 29.9% in England). The percentage of infants with any unplanned hospital contact was similar between countries (42.9% in Ontario, 41.6% in England) as was mortality (0.05% in Ontario, 0.06% in England). Infants attending ED were less likely to be admitted in Ontario (7.3% vs 26.2%), but those who were admitted were more likely to stay for ≥1 night (94.0% vs 55.2%). The strongest risk factors for admission were completed weeks of gestation (adjusted OR for 34–36 weeks vs 39+ weeks: 2.44; 95% CI 2.29 to 2.61 in Ontario and 1.66; 95% CI 1.62 to 1.70 in England) and young maternal age.

**Conclusions:**

Children attending ED in England were much more likely to be admitted than those in Ontario. The tendency towards more frequent, shorter admissions in England could be due to more pressure to admit within waiting time targets, or less availability of paediatric expertise in ED. Further evaluations should consider where best to focus resources, including in-hospital, primary care and paediatric care in the community.

## Background

Paediatric emergency admissions in England have risen by a third over the past decade and are continuing to rise.^1 2^ For serious conditions, admissions are necessary and appropriate. However, the majority of unplanned admissions within the first year of life are for minor conditions, particularly minor infections, which could be treated outside hospital.^1 3^ Responding to infant illness in ways that avoid emergency admission could help reduce inconvenience to families (eg, emotional distress, interruption of work or school) and the risk of iatrogeny (eg, hospital-acquired infection or medical errors). However, there is a lack of evidence on the best approaches to reducing unplanned infant admissions.^4^

Analyses of trends over time in admission rates have provided important insights into how changes to policy influence paediatric healthcare use (eg, how UK primary care policy reforms aimed at improving working conditions for physicians and outcomes for patients are related to increasing rates of short-stay paediatric admissions).^1 5^ Comparisons of healthcare use in different settings provide further opportunities to identify determinants of variation in service use.^6^ Specifically, Ontario (Canada’s largest province) and England have similar cultural and environmental risk factors and similar levels of child poverty.^7^ Both countries offer universal healthcare systems, with no user fees at the point of care. General practitioners (GPs) also operate similar gatekeeper functions in both countries, referring families to hospital where appropriate. However, important organisational differences between these jurisdictions provide an opportunity to understand how different policies and service provision may contribute to paediatric acute healthcare use.

For example, although most of the Ontario population has a primary care provider, access to a doctor out of hours without attending emergency department (ED) is very low in comparison to other OECD (Organisation for Economic Co-operation and Development) countries.^8^ Primary care reform in Ontario has attempted to improve access through incentives aimed at general/family practitioners, although children also receive primary care from paediatricians (predominantly in urban centres).^9^ There are also differences in postnatal support during early infancy: all families in England are supported by home visiting by qualified midwives and health visitors, whereas follow-up is less well established in Ontario, where only 9% of pregnant mothers are cared for by a midwife and less than 35% of low-risk newborns receive the recommended follow-up visit in the first week of life.^10–12^ While Canada has a well-established history of paediatric emergency medicine (PEM) in tertiary centres and a system of consultant paediatricians in the ED in large community hospitals,^13^ consultant PEM provision in the UK varies regionally, and is only recommended for emergency care settings seeing more than 16 000 children per year (around half of EDs in the UK).^14^ Finally, ED wait time targets also differ between countries: in England, 95% of patients attending an ED should be seen, treated, admitted or discharged in under 4 hours (98% prior to 2010), whereas in Ontario, 90% of ED visits for patients with only minor or uncomplicated conditions are expected to be completed within 4 hours.

We performed an in-depth comparison of emergency hospital use during infancy, a time of high need for acute care, within which admissions may be avoidable, and a time when neonatal morbidity and social risk factors may be especially important. We evaluated both inpatient admissions and ED visits in order to gain an overall picture of hospital contact. We aimed to identify differences in patterns of hospital use and maternal and neonatal risk factors, based on standardised birth cohorts of healthy populations, derived from linked administrative hospital data.

## Methods

### Data sources

We extracted data for Ontario from linked population-based administrative databases at the Institute for Clinical and Evaluative Sciences in Toronto.^15^ Eligible mothers and infants were identified from the Registered Persons Database, which holds information on all Ontario residents (currently over 13 million) with a provincial health card number. Linked maternal and newborn health records were extracted from the MOMBABY dataset, which provides information on all births in hospitals in Ontario and is linked using a unique health card number. Inpatient admissions and ED visits were extracted for all hospitals providing acute inpatient facilities in Ontario from the Canadian Institute of Health Information Discharge Abstract Database and the National Ambulatory Care Reporting System Database.

For England, data were extracted from Hospital Episode Statistics (HES).^16^ HES is an administrative database holding detailed information for all admissions to National Health Service (NHS) hospitals in England. Maternal and baby birth characteristics were linked using non-identifiable clinical and demographic information available on the main HES record (including admission dates, postcode district and GP practice) and delivery information contained in the ‘baby tail’ (including gestational age, birth weight and mode of delivery). Full details of the linkage have been published elsewhere.^17^ Inpatient admissions and ED visits were extracted from the Admitted Patient Care dataset and the Accident & Emergency dataset.

For both countries, hospital records are collected for reimbursement purposes, and are encoded, allowing admissions for the same patient to be tracked over time. Admission records allow the entry of multiple fields of clinical diagnoses (24 fields in Ontario, 20 in England) coded using the International Statistical Classification of Diseases and Related Health Problems 10th Revision (ICD-10).

### Study population

Our study population comprised newborns alive at postnatal discharge. Newborns <34 weeks’ gestation were excluded due to their more complex health needs (1.8% of births in Ontario and 1.7% of births in England). Since we knew that gestational age distributions differed between countries, and expected that gestational age would be strongly predictive of healthcare contact, we stratified results by late-preterm (34–36 weeks), early-term (37–38 weeks) or full-term (39+ weeks) births.

We focused our analysis on data on births from April 2010 onwards, as ED data were not available in England prior to this time. Follow-up data were complete until March 2014 for all births before 1 March 2013. We therefore included births between 1 April 2010 and 1 March 2013. We also plotted trends in admissions from April 2005 onwards.

For mothers with multiple deliveries during the study period, we randomly selected one delivery for inclusion in analyses, to avoid clustering of outcomes by mother. We excluded infants with missing gestation or birth weight or suspected coding errors (birth weight >4 SDs from the average according to published reference values for each country).^18 19^ For England, the percentage of births excluded due to missing values ranged from 10% in 2010/2011 to 7% in 2012/2013. The level of missing data in the Ontario data was negligible (<1%).

### Outcomes

All outcomes were captured in hospital records up to 12 months from postnatal discharge. The primary outcome was the percentage of infants with one or more unplanned admissions. Admissions were defined as unplanned based on the method of admission coded within the hospital record, and were defined as episodes of care starting more than 2 days following the end of a previous admission. Transfers between hospitals were not counted as readmissions.

We also evaluated a number of secondary outcomes. We compared ED visits to any hospital (with or without subsequent admission). For England, ED data were provided for all NHS hospitals included in the study population (n=152), but reliable ED diagnosis data were not available.

To measure overall hospital contact, we compared the percentage of infants with any emergency contact (unplanned admission or ED) and the total number of inpatient days from unplanned admissions (both including and excluding the birth episode). We also compared postdischarge deaths.

A further secondary outcome was overnight admissions, where infants were admitted and discharged on different days (ie, admissions starting and ending on the same day were excluded). Finally, we compared the number of unplanned admissions for different diagnosis groups, based on the 10 most frequently occurring ICD-10 code groups in the main diagnosis fields for admission records in each country.

### Risk factors

Gestational age in completed weeks was obtained from the linked maternal-baby data, based on best estimates from menstrual dates or ultrasound. Small or large for gestation (<10th or >90th percentile of birth weight for gestation) was derived from national birth weight percentiles.^18 19^ Delivery by caesarean section, sex, multiple birth, admission to neonatal intensive care, season of discharge and maternal age were considered as additional covariates. Measures of deprivation were obtained via postal code of residence mapped to neighbourhood income quintile in Ontario, and Index of Multiple Deprivation (IMD) for England.^20^ Based on code lists used in previous studies, we also derived a number of pregnancy, delivery and neonatal risk factors using ICD-10 codes recorded in any diagnosis field during the birth episode or pregnancy (see table 1 and online supplementary appendix table 1 for code lists).^21 22^ We explored trends by including a variable for quarterly trend, that is, taking values of 1–12 for each quarter year of postnatal discharge between April 2010 and March 2013. We also plotted outcomes by quarter. Due to the size of the datasets, p values for differences in outcomes between countries were not presented, as all comparisons were statistically significant at the 95% confidence level.

10.1136/bmjqs-2016-006253.supp1 Supplementary Appendix 1

**Table 1:** Characteristics of mothers and live births between 2010 and 2013 by country

		Ontario (n=253 930)		England (n=1 361 128)	
		N	%	N	%
Gestational age group	Full term (39+ weeks)	170 445	67.1	1 047 532	77.0
	Early term (37–38 weeks)	69 349	27.3	250 029	18.4
	Late preterm (34–36 weeks)	14 136	5.6	63 567	4.7
Birth weight	<1500	140	0.1	1125	0.1
	1500–<2500	10 817	4.3	63 319	4.7
	2500–<4000	216 777	85.4	1 141 544	83.9
	4000+	26 196	10.3	155 140	11.4
Size for gestation	Small (<10th percentile)	25 825	10.2	120 322	8.8
	Normal	204 271	80.4	1 103 893	81.1
	Large (>10th percentile)	23 834	9.4	136 913	10.1
Maternal age (years)	<20	9439	3.7	78 659	5.8
	20–24	31 974	12.6	255 986	18.9
	25–29	70 759	27.9	375 835	27.7
	30–34	85 016	33.5	385 700	28.4
	35–39	45 448	17.9	207 269	15.3
	40+	11 291	4.4	54 021	4.0
Female infant		123 937	48.8	663 798	48.8
Multiple birth		4618	1.8	19 973	1.5
Primiparous mother		133 167	52.4	661 402	48.6
Income/deprivation quintile	Most deprived: 1	55 945	22.0	266 211	19.8
	2	50 818	20.0	269 664	20.1
	3	50 472	19.9	268 799	20.0
	4	51 914	20.4	270 125	20.1
	Least deprived: 5	39 121	15.4	267 068	19.9
Newborn length of stay (days)	<2	97 728	38.5	727 461	53.5
	2–6	147 294	58.0	577 452	42.4
	7+	8908	3.5	56 215	4.1
Caesarean section		74 067	29.2	319 194	23.5
Neonatal ICU		26 463	10.4	148 957	10.9
Delivery risk factor (Any)		33 959	13.4	37 062	10.1
	Hypoxia	1242	0.5	78 894	5.8
	Amniotic fluid embolism	14	0.0	18	0.0
	Placental transfusion syndrome	61	0.0	203	0.0
	Umbilical cord prolapse	27 780	10.9	19 374	1.4
	Chorioamnionitis	2270	0.9	2412	0.2
	Fetal haemorrhage	358	0.1	5783	0.4
	Birth trauma	1748	0.7	39 313	2.9
	Complications of delivery	1688	0.7	18 244	1.3
	Umbilical cord problem	888	0.3	2871	0.2
Pregnancy risk factor (Any)		31 103	12.2	49 687	11.0
	Previous intrauterine fetal death	21	0.0	139	0.0
	Eclampsia	2858	1.1	31 084	2.3
	Gestational hypertension	11 911	4.7	79 576	5.9
	Diabetes in pregnancy	15 405	6.1	53 170	3.9
	Placental abruption or infarction	3211	1.3	14 468	1.1
	Uterine rupture	226	0.1	679	0.1
Neonatal medical condition (Any)		10 478	4.1	59 818	4.4
	Congenital anomaly	5884	2.3	24 763	1.8
	Perinatal infection	1876	0.7	29 093	2.1
	Neonatal abstinence syndrome	1024	0.4	2504	0.2
	Respiratory distress syndrome	2924	1.2	9405	0.7

ICU, intensive care unit.

### Statistical analysis

The risk of one or more admissions or ED visits was modelled using multilevel logistic regression, allowing for clustering within healthcare provider at postnatal discharge (hospital for Ontario and NHS Trust for England were included as random effects). In models for admission or ED visits, infants who died within 12 months post discharge were treated as having the outcome. Adjusted ORs (aOR) were used to compare the risk of admission or ED visit according to common risk factors within each country. To allow us to assess the impact of common risk factors on outcomes in each country, all predefined variables were retained in models, irrespective of statistical significance.

Sharing of record-level data outside of each country was not permitted, and we could not incorporate a ‘country’ variable within our models. Instead, we separately modelled data within each country. However, this allowed us to explore the impact of risk factors within each country, without making assumptions about similarity of coding. For example, deprivation was derived from income quintile in Ontario (ie, a direct measure of income) and IMD in England (ie, a measure of material deprivation). Separate models allowed us to compare the impact of being in the lowest versus highest quintile within each country. Analyses were performed in Stata V.14.^23^

## Results

A total of 253 930 (Ontario) and 1 361 128 (England) mother–baby dyads were included in the study (table 1). Characteristics of mothers and infants were broadly similar between countries, with a few exceptions: Ontario had a greater proportion of early-term and late-preterm births, more births by caesarean section and fewer young mothers (table 1).

**Figure 1:**
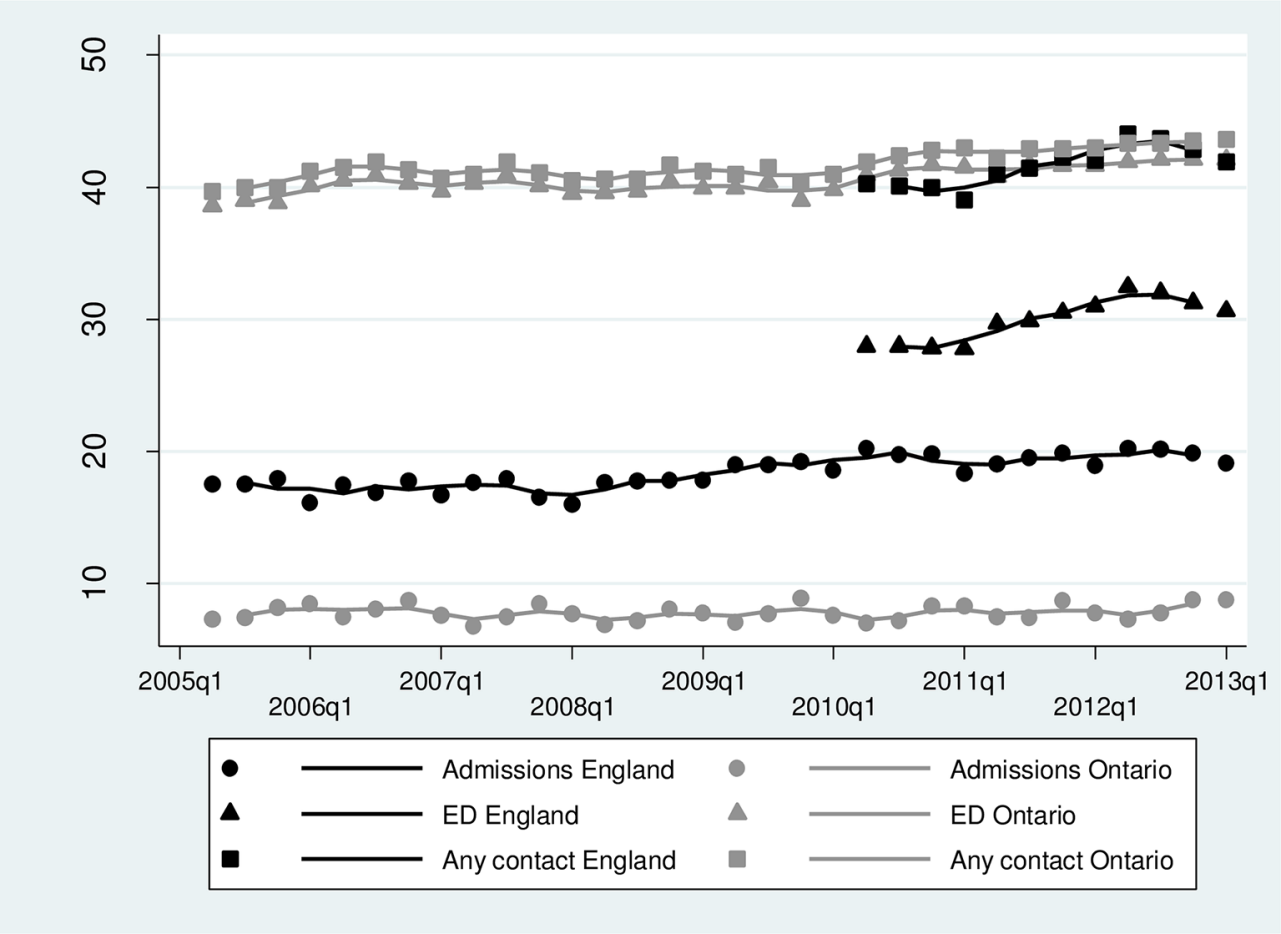
Trends in the percentage of infants with ≥1 unplanned admission, ED visit or any contact (admission or ED) within 12 months of postnatal discharge. Symbols=observed rates; lines=three-quarter moving average. ED, emergency department.

The percentage of infants with at least one unplanned admission within 12 months of postnatal discharge was substantially lower in Ontario (7.9% vs 19.6% in England, table 2 and figure 1), while the percentage of infants attending ED but not being admitted was much higher (39.8% vs 29.9% in England). ED visits were much less likely to result in an admission in Ontario (7.3% of ED visits resulted in an admission vs 26.2% in England), and were of a slightly longer duration (median 2 hours 5 min vs 1 hour 49 min in England). The percentage of unplanned admissions admitted via ED was similar in both countries (66.1% in Ontario, 67.9% in England), while the percentage recorded as being admitted via a physician or GP was slightly lower in Ontario (23.6% vs 29.1% in England).

**Table 2:** Infant outcomes within 12 months post discharge, by gestational age group and country, for births between 2010 and 2013

		N (%) infants with ≥1 event to 12 months postnatal discharge						Mean inpatient days per infant to 12 months postnatal discharge	
		All-cause admission	Overnight admission*	ED visit not admitted	Any ED visit	Any contact (admission or ED)	Mortality	From postnatal discharge	From day of birth
Total	Ontario n=253 930	20 016	18 954	101 060	105 661	108 903	139	0.3	2.7
		(7.9)	(7.5)	(39.8)	(41.6)	(42.9)	(0.05)		
	England n=1 361 128	266 771	160 690	407 331	491 991	565 896	837	0.6	2.8
		(19.6)	(11.8)	(29.9)	(36.2)	(41.6)	(0.06)		
Full term (39+ weeks)	Ontario n=170 445	11 178	10 524	66 634	69 351	70 910	67	0.2	2.2
		(6.6)	(6.2)	(39.1)	(40.7)	(41.6)	(0.04)		
	England n=1 047 532	189 921	112 235	307 362	368 752	421 097	506	0.5	2.4
		(18.1)	(10.7)	(29.3)	(35.2)	(40.2)	(0.05)		
Early term (37–38 weeks)	Ontario n=69 349	6929	6601	28 270	29 805	31 102	54	0.4	2.8
		(10.0)	(9.5)	(40.8)	(43.0)	(44.9)	(0.08)		
	England n=250 029	58 343	36 309	79 005	96 726	113 116	215	0.7	3.3
		(23.3)	(14.5)	(31.6)	(38.7)	(45.3)	(0.09)		
Late preterm (34–36 weeks)	Ontario n=14 136	1909	1829	6505	6891	6156	18	0.6	7.7
		(13.5)	(12.9)	(46.0)	(48.8)	(43.6)	(0.19)		
	England n=63 567	18 507	12 146	20 964	26 513	31 683	116	1.2	8.2
		(29.1)	(19.1)	(33.0)	(41.7)	(49.9)	(0.18)		

*All-cause admission excluding those admitted and discharged on the same day.

ED, emergency department.

Overall hospital contact during infancy was similar between countries: the percentage of infants with either unplanned admission or ED attendance was 42.9% in Ontario and 41.6% in England. Mortality was also the same in both countries (0.05% in Ontario, 0.06% in England).

Infants who were admitted were more likely to stay for ≥1 night in Ontario (94.0% vs 55.2% in England) and have longer admissions (median 3.9 days compared with 2.2 days in England). However, due to the greater number of admissions, mean inpatient days per infant post discharge in England were almost double those in Ontario (table 2).

Rates of unplanned admissions and ED visits were increasing over time in both countries, but to a greater extent in England (figure 1, table 3). Other risk factors for infant admission were of similar magnitudes in both countries, with the exception of caesarean section and newborn length of stay (table 3). Gestational age at birth was the most important risk factor for infant admission: aORs for infants born late preterm (34–36 weeks) were 2.44 (95% CI 2.29 to 2.61) in Ontario and 1.66 (95% CI 1.62 to 1.70) in England, compared with full-term babies (39+ weeks) (table 3). Young maternal age (<20 years) was highly predictive of infant admission (aOR 1.36, 95% CI 1.26 to 1.46 in Ontario; 1.49, 95% CI 1.46 to 1.52 in England; table 3) and ED visits (see online supplementary appendix table 2). Deprivation was also an important risk factor in both countries, particularly for ED visits (see online supplementary appendix table 2).

**Table 3:** Risk factors for unplanned hospital admission in infants in England and Ontario, 2010–2013

		Ontario		England	
		OR (95% CI)	p Value	OR (95% CI)	p Value
Gestational age at birth (weeks)	Full term (39+)	1	<0.001	1	<0.001
	Early term (37-38)	1.64 (1.59 to 1.70)		1.39 (1.37 to 1.40)	
	Late preterm (34-36)	2.44 (2.29 to 2.61)		1.66 (1.62 to 1.70)	
Newborn length of stay (days)	<2	1	0.024	1	<0.001
	2–6	1.02 (0.98 to 1.06)		1.08 (1.07 to 1.10)	
	7–13	0.91 (0.83 to 1.00)		1.38 (1.34 to 1.41)	
Size for gestation	Small (<10th percentile)	1.02 (0.98 to 1.03)	<0.001	1.07 (1.05 to 1.09)	<0.001
	Normal	1		1	
	Large (>90th percentile)	1.06 (1.01 to 1.12)		1.00 (0.98 to 1.01)	
Maternal age (years)	≤19	1.36 (1.26 to 1.46)	<0.001	1.49 (1.46 to 1.52)	<0.001
	20–24	1.18 (1.12 to 1.24)		1.20 (1.19 to 1.22)	
	25–29	1		1	
	30–34	0.96 (0.93 to 1.00)		0.90 (0.89 to 0.91)	
	35–39	0.95 (0.91 to 1.00)		0.83 (0.82 to 0.84)	
	≥40	0.95 (0.88 to 1.03)		0.82 (0.80 to 0.84)	
Female sex		0.79 (0.76 to 0.81)	<0.001	0.80 (0.80 to 0.81)	<0.001
Primiparous mother		0.92 (0.89 to 0.95)	<0.001	0.89 (0.88 to 0.90)	<0.001
Multiple birth		0.66 (0.59 to 0.74)	<0.001	0.81 (0.78 to 0.84)	<0.001
Deprivation quintile	Most deprived	1.12 (1.06 to 1.17)	<0.001	1.03 (1.01 to 1.04)	<0.001
	2	1.05 (1.00 to 1.10)		1.01 (1.00 to 1.03)	
	3	1.06 (1.00 to 1.11)		1.01 (0.99 to 1.02)	
	4	1.06 (1.00 to 1.11)		0.99 (0.98 to 1.00)	
	Most affluent	1		1	
Caesarean section		0.77 (0.74 to 0.80)	<0.001	1.04 (1.02 to 1.05)	<0.001
Admission to neonatal intensive care		1.03 (0.98 to 1.09)	0.232	1.03 (1.01 to 1.05)	<0.001
Season of discharge	January to March	1	<0.001	1	<0.001
	April to June	0.89 (0.85 to 0.93)		1.08 (1.07 to 1.10)	
	July to September	0.91 (0.87 to 0.95)		1.08 (1.07 to 1.10)	
	October to December	1.06 (1.02 to 1.11)		1.08 (1.06 to 1.09)	
Quarter year of discharge		1.01 (1.01 to 1.02)	<0.001	1.00 (1.00 to 1.00)	<0.001
Perinatal infection		1.09 (0.94 to 1.28)	0.249	1.09 (1.06 to 1.12)	<0.001
Prematurity related risk factor		0.87 (0.77 to 0.99)	0.033	1.01 (0.96 to 1.06)	0.715
Neonatal medical condition		1.82 (1.68 to 1.97)	<0.001	1.84 (1.79 to 1.89)	<0.001
Pregnancy risk factor		1.07 (1.02 to 1.12)	0.003	1.00 (.099 to 1.03)	0.525
Delivery risk factor		1.01 (0.97 to 1.06)	0.605	1.01 (1.00 to 1.03)	0.112

**Figure 2:**
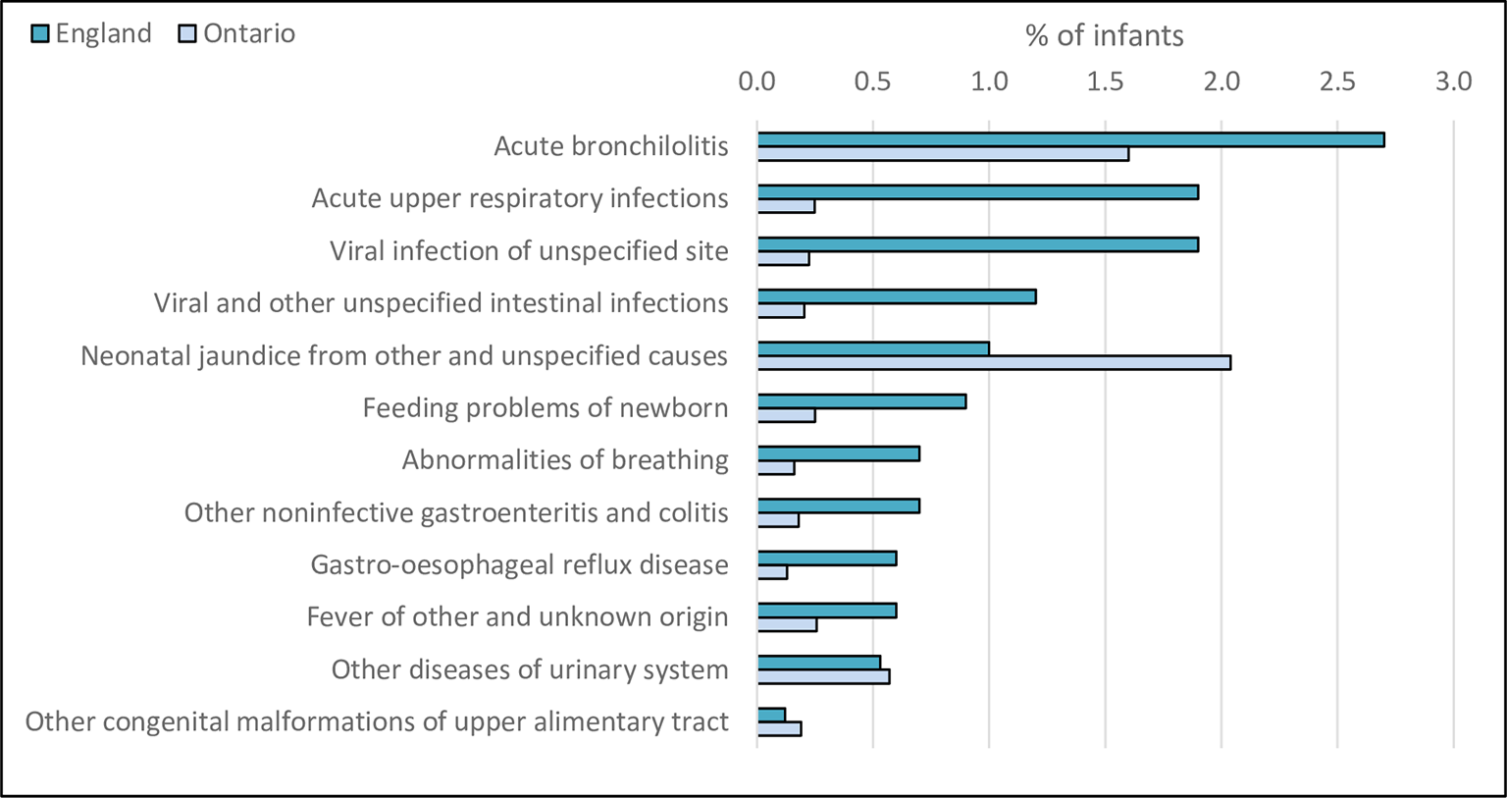
Most frequently occurring diagnoses from unplanned admissions within 12 months of postnatal discharge.

The most frequently occurring admission diagnoses in both countries were acute upper respiratory tract infections, bronchiolitis and viral infections (figure 2). Despite lower admission rates overall, the percentage of infants readmitted with neonatal jaundice was substantially higher in Ontario compared with England.

## Discussion

Our study illustrates the complexities of international comparisons of hospital use. Despite similar health service provision and standardised cohorts of healthy babies in our study populations, focusing solely on the inpatient setting would have revealed substantially higher levels of emergency hospital use in England compared with Ontario. However, by considering both inpatient admissions and ED visits, we showed that overall hospital contact (either ED or admission) and total inpatient days from birth to 12 months were similar across countries. We found different patterns of hospital use that suggest variation in admission thresholds between countries: in Ontario, infants seen in EDs were much less likely to be admitted compared with those in England, while infants admitted in England were more often discharged on the same day as admission.

Infant admission rates observed in our study were low for Ontario (7.9%) and relatively high for England (19.6%) compared with those reported in Australia (15%–20%) and the USA (8%–12%).^24–27^ There are various contributing factors that might explain the differences in hospitalisation patterns between the two countries in our study, including differences in primary or secondary care practice, or in the underlying population.^28^ Although we could not directly assess the role of primary care in either country, data suggested that the percentage of infants admitted via a physician or GP was slightly higher in England (29.1% vs 23.6%). Our study populations reflect official statistics published in each jurisdiction and demonstrate some important differences between populations: Ontario has fewer young mothers,^29 30^ more births by caesarean section^31 32^ and a greater proportion of early-term births.^33 34^ Although the greater number of infants born early in Ontario was not reflected in an overall higher admission rate during infancy, jaundice (an early outcome associated with early-term birth) appeared to be a greater problem in Ontario.^24 35^

Another possible explanation for differences in admission rates between countries is the availability of trained emergency paediatricians in ED. Although we could not directly measure this in our study, consultant PEM is better established in Canada than England.^13 14^ Only one study has evaluated the effectiveness of PEM provision, finding that increased consultant provision was associated with lower admission rates.^36^ Further research is needed to determine whether increasing emergency paediatrician provision in EDs in England could reduce the numbers of infants admitted for short-stay emergency admissions, or whether this could better be achieved through more efficient management of children with acute illnesses within the community.^2 5 28^

Differences in ED wait time targets between countries may also have contributed to differences in admission rates. Implementation of ED waiting time targets in the NHS has resulted in increased pressure to admit, and a marked increase in admissions just before the 4-hour cut-off.^37^ Targets may also have altered health-seeking behaviours, motivating families to seek ED review rather than wait for an appointment with a GP.^38^ This was reflected in increasing ED attendance rates over time in both countries. In our study, ‘zero-day’ admissions were much more common in England (45% of admissions were admitted and discharged on the same day compared with 6% in Ontario). Pressure to admit from ED explains some but not all of this difference: in England, 50% of admissions with a preceding ED visit were discharged on the same day, compared with 38% of admissions without a preceding ED visit. Greater travel distances in Ontario may also have a role in explaining the greater proportion of overnight admissions. For example, clinicians in Ontario may choose to admit an infant overnight rather than send them on a long late-night journey. Data were not available to test this within our study.

Further research is needed to determine whether service provision is more effective in Ontario or England. Hospital admission increases exposure to nosocomial infection, medical error and adverse drug reactions, and can contribute to psychological distress, disruption, or economic loss for children and/or their families.^39–41^ Reducing unplanned admissions has the potential to improve quality of life for children and their families, as well as alleviating pressure on hospital resources, and is recognised as an important indicator of quality by the NHS Outcomes Framework.^42^ However, there is a complex relationship between relevant outcomes for children and their families, and primary care access, ED attendances and short-stay admissions, and it is unclear where best to focus investment.^38 43 44^

As with all studies based on data collected for purposes other than research, careful interpretation of observed differences is required, taking into account the implications of any variation in data quality or coding practices.^45^ A limitation of this study was the level of missing data on gestational age or birth weight in the English data, which led to the exclusion of some records. However, the representativeness of the study population that did have complete data, means that missing data are unlikely to have led to substantial biases. A lack of validation of risk-factor codes could have led to some misclassification, for example, on capturing congenital anomalies or admission to neonatal intensive care. Although gestational age should be recorded in the same way in both countries (estimated from either ultrasound or last menstrual period), there may be differences in derivation between countries that could have led to misclassification. Since measures of socioeconomic status were different in each country, adjustments within countries were made assuming that differences between quintiles (of either income or multiple deprivation) were similar. However, recording of admission and ED patterns, based on event data, is likely to be accurate. A further limitation of our study was a lack of information on general practice and out-of-hospital care. Health-seeking behaviour appeared similar between countries, with the proportion of families visiting EDs being slightly higher in Ontario. We were unable to assess staffing provision or supply of hospital beds.

Administrative data provide a powerful tool for determining how service use varies between countries with similar cultural and environmental risk factors, identifying policy areas that could be compared, and generating hypotheses about how organisational-level factors and service provision contribute to outcomes.^6 46^ Our study in particular demonstrates the importance of incorporating detailed information on both maternal and baby characteristics and complete healthcare trajectories for exploring variation in emergency care.^17^ In Ontario, linkage was facilitated by a population spine (the Registered Person database). No such spine currently exists for England, although linkage of prospective maternity data is being developed by NHS Digital for the Maternity and Children’s Dataset.^17 47^ Further research on the burden of recurrent admissions in both countries would support policymakers to consider the comparative effectiveness and cost-effectiveness of focusing paediatric expertise in ED versus inpatient settings and primary care.

## References

[1] Saxena S, Bottle A, Gilbert R, et al Increasing short-stay unplanned hospital admissions among children in England; time trends analysis '97-'06. PLoS One 2009;4:e7484 10.1371/journal.pone.0007484 19829695 PMC2758998

[2] Gill PJ, Goldacre MJ, Mant D, et al Increase in emergency admissions to hospital for children aged under 15 in England, 1999-2010: national database analysis. Arch Dis Child 2013;98:328–34. 10.1136/archdischild-2012-302383 23401058

[3] Wijlaars LP, Hardelid P, Woodman J, et al Who comes back with what: a retrospective database study on reasons for emergency readmission to hospital in children and young people in England. Arch Dis Child 2016;101:714–8. 10.1136/archdischild-2015-309290 27113555

[4] Coon JT, Martin A, Abdul-Rahman AK, et al Interventions to reduce acute paediatric hospital admissions: a systematic review. Arch Dis Child 2012;97:304–11. 10.1136/archdischild-2011-301214 22294664

[5] Cecil E, Bottle A, Sharland M, et al Impact of UK primary care policy reforms on short-stay unplanned hospital admissions for children with primary care-sensitive conditions. Ann Fam Med 2015;13:214–20. 10.1370/afm.1786 25964398 PMC4427415

[6] Australian Institute of Health and Welfare. A working guide to international comparisons of health. Canberra: AIHW, 2012 PHE 159.

[7] Menchini L, Chzhen Y, Main G, et al Relative income poverty among children in rich countries. 2012.

[8] Osborn R, Moulds D, Schneider EC, et al Primary care physicians in ten countries report challenges caring for patients with complex health needs. Health Aff 2015;34:2104–12. 10.1377/hlthaff.2015.1018 26643631

[9] Guttmann A, Gandhi S, Hanvey P, et al Section 3: primary Health Care Services for Children and Youth in Canada: access, Quality and structure: In The Health of Canada’s Children and Youth: A CICH Profile, 2017.

[10] Society CP, detection G Guidelines for detection, management and prevention of hyperbilirubinemia in term and late preterm newborn infants (35 or more weeks' gestation) - summary. Paediatr Child Health 2007;12:401–7. 19030436 10.1093/pch/12.7.613 PMC2528770

[11] Darling EK, Ramsay T, Manuel D, et al Association of universal bilirubin screening with socioeconomic disparities in newborn follow-up. Acad Pediatr 2017;17:135–43. 10.1016/j.acap.2016.07.009 27497623

[12] Kurtz Landy C, Sword W, Ciliska D Urban women’s socioeconomic status, health service needs and utilization in the four weeks after postpartum hospital discharge: findings of a Canadian cross-sectional survey. BMC Health Serv Res 2008;8:1–9. 10.1186/1472-6963-8-203 18834521 PMC2570364

[13] McGillivray D, Jarvis A A history of paediatric emergency medicine in Canada. Paediatr Child Health 2007;12:453–6. 19030405 10.1093/pch/12.6.453 PMC2528768

[14] Davies FC, Newton T Paediatric emergency medicine consultant provision in the UK: are we there yet? Arch Dis Child 2015;100:1016–7. 10.1136/archdischild-2015-308952 26194358

[15] Williams TA, Dobb GJ, Finn JC, et al Data linkage enables evaluation of long-term survival after intensive care. Anaesth Intensive Care 2006;34:307–15. 16802482 10.1177/0310057X0603400316

[16] Herbert A, Wijlaars L, Zylbersztejn A, et al Data Resource Profile: Hospital Episode Statistics Admitted Patient Care (HES APC). Int J Epidemiol. In Press 2017 10.1093/ije/dyx015 PMC5837677 28338941

[17] Harron K, Gilbert R, Cromwell D, et al Linking data for mothers and babies in de-identified electronic health data. PLoS One 2016;11:e0164667 10.1371/journal.pone.0164667 27764135 PMC5072610

[18] Kramer MS, Platt RW, Wen SW, et al A new and improved population-based Canadian reference for birth weight for gestational age. Pediatrics 2001;108:e35 10.1542/peds.108.2.e35 11483845

[19] Cole TJ, Statnikov Y, Santhakumaran S, et al Birth weight and longitudinal growth in infants born below 32 weeks' gestation: a UK population study. Arch Dis Child Fetal Neonatal Ed 2014;99:F34–40. 10.1136/archdischild-2012-303536 23934365 PMC3888637

[20] Office of the Deputy Prime Minister The English Indices of Deprivation 2004, 2003.

[21] Unterscheider J, Daly S, Geary MP, et al Optimizing the definition of intrauterine growth restriction: the multicenter prospective PORTO Study. Am J Obstet Gynecol 2013;208:290 e1-90.e6 10.1016/j.ajog.2013.02.007 23531326

[22] Ray JG, Vermeulen MJ, Schull MJ, et al Cardiovascular health after maternal placental syndromes (CHAMPS): population-based retrospective cohort study. Lancet 2005;366:1797–803. 10.1016/S0140-6736(05)67726-4 16298217

[23] Stata Statistical Software: release 14[program]. College Station, TX StataCorp LP 2015.

[24] Lain SJ, Nassar N, Bowen JR, et al Risk factors and costs of hospital admissions in first year of life: a population-based study. J Pediatr 2013;163:1014–9. 10.1016/j.jpeds.2013.04.051 23769505

[25] Bird TM, Bronstein JM, Hall RW, et al Late preterm infants: birth outcomes and health care utilization in the first year. Pediatrics 2010;126:e311–19. 10.1542/peds.2009-2869 20603259

[26] McLaurin KK, Hall CB, Jackson EA, et al Persistence of morbidity and cost differences between late-preterm and term infants during the first year of life. Pediatrics 2009;123:653–9. 10.1542/peds.2008-1439 19171634

[27] Colvin L, Bower C A retrospective population-based study of childhood hospital admissions with record linkage to a birth defects registry. BMC Pediatr 2009;9:1–13. 10.1186/1471-2431-9-32 19426556 PMC2692976

[28] Ogilvie D Hospital based alternatives to acute paediatric admission: a systematic review. Arch Dis Child 2005;90:138–42. 10.1136/adc.2003.035543 15665164 PMC1720277

[29] Office for National Statistics Birth Summary Tables - England and Wales.

[30] Statistics Canada. CANSIM Table 102-4503 - Live births, by age of mother, Canada, provinces and territories, Annual.

[31] The Information Centre. Hospital Episode Statistics: nhs Maternity Statistics, 2010-2011, 2011.

[32] Public Health Agency of Canada. perinatal health indicators for Canada A report from the Canadian Perinatal Surveillance System, 2013.

[33] Office for National Statistics Gestation-specific Infant Mortality, 2012, 2014.

[34] Statistics Canada. CANSIM Table 102-4512 - Live births, by weeks of gestation and sex, Canada, provinces and territories, Annual.

[35] Saigal S, Doyle LW An overview of mortality and sequelae of preterm birth from infancy to adulthood. Lancet 2008;371:261–9. 10.1016/S0140-6736(08)60136-1 18207020

[36] Geelhoed GC, Geelhoed EA Positive impact of increased number of emergency consultants. Arch Dis Child 2008;93:62–4. 10.1136/adc.2007.122531 17768147

[37] Mooney H Admissions from emergency departments rise as four hour target approaches. BMJ 2009;339:b4931 10.1136/bmj.b4931

[38] Cowling TE, Soljak MA, Bell D, et al Emergency hospital admissions via accident and emergency departments in England: time trend, conceptual framework and policy implications. J R Soc Med 2014;107:432–8. 10.1177/0141076814542669 25377736 PMC4224646

[39] Rennick JE, Johnston CC, Dougherty G, et al Children’s psychological responses after critical illness and exposure to invasive technology. J Dev Behav Pediatr 2002;23:133–44. 10.1097/00004703-200206000-00002 12055495

[40] Forgey M, Bursch B Assessment and management of pediatric iatrogenic medical trauma. Curr Psychiatry Rep 2013;15:340 10.1007/s11920-012-0340-5 23307562

[41] Flores G Preventing hospitalisations for children. Lancet 2005;365:201–2. 10.1016/S0140-6736(05)70130-6 15652590

[42] Clinical Indicators Team NHS Digital NHS Outcomes Framework: Quarterly Publication 2017, 2017.

[43] Cecil E, Bottle A, Cowling TE, et al Primary Care access, Emergency Department Visits, and Unplanned Short Hospitalizations in the UK. Pediatrics 2016;137:e20151492 10.1542/peds.2015-1492 26791971

[44] Guttmann A International perspectives on primary care access, equity, and outcomes for children. Pediatrics 2016;137:e20154163 10.1542/peds.2015-4163 26791969

[45] Benchimol EI, Smeeth L, Guttmann A, et al The REporting of studies Conducted using Observational Routinely-collected Health Data (RECORD) Statement. PLoS Med 2015;12:e1001885 10.1371/journal.pmed.1001885 26440803 PMC4595218

[46] Saigal S, den Ouden L, Wolke D, et al School-age outcomes in children who were extremely low birth weight from four international population-based cohorts. Pediatrics 2003;112:943–50. 10.1542/peds.112.4.943 14523190

[47] Health and Social Care Information Centre. Maternity Services Data Set (MSDS) Data Model v1.5. Secondary Maternity Services Data set (MSDS) Data Model v1.5. 2015 http://www.hscic.gov.uk/media/11885/Maternity-Data-Model-v15/pdf/Maternity_Services_Data_Set_Data_Model_v1.5.pdf.

