# Supplementary material for: International comparison of emergency hospital use for infants: data linkage cohort study in Canada and England
Source: BMJ Qual Saf. 2017 Jun 12;27(1):31–9. doi: 10.1136/bmjqs-2016-006253 (PMC5750429; doi:10.1136/bmjqs-2016-006253)
Supplement: Supplementary Appendix 1 [file bmjqs-2016-006253supp001.docx]

## **SUPPLEMENTARY APPENDIX**

**Appendix Table 1: ICD 10 code lists for pregnancy, delivery and neonatal risk factors**

| **Group** | **Description** | **ICD10 codes** |
| --- | --- | --- |
| Pregnancy risk factor | Intrauterine fetal death | O364, P95 |
|  | Eclampsia | O14,O15 |
|  | Gestational hypertension | O13,O16 |
|  | Placental abruption or infarction | O45,O431, O438, O439 |
|  | Uterine rupture | O710, O711 |
|  | Diabetes in pregnancy | P700, O24, E10-E14 |
| Delivery risk factor | Birth trauma | P10-P15 |
|  | Complications of delivery | P03 |
|  | Hypoxia | P20-P21 |
|  | Amniotic fluid embolism | O881 |
|  | Chorioamnionitis | O411 P027-P029 |
|  | Umbilical cord problem | P020, P024-P026 |
|  | Fetal hemorrhage | P50, P51, P53, P54 |
|  | Maternal hemorrhage | O430 |
|  | Umbilical cord prolapse | O69 |
| Neonatal medical condition | Congenital anomalies | Q00-Q07, Q10.4, Q10.7, Q11-Q12, Q13.0-Q13.4, Q13.8, Q13.9, Q14-Q16, Q20-Q26, Q18.8, Q30-Q37, Q38.0, Q38.3, Q38.4, Q38.6-Q38.8, Q39, Q40.2, Q40.3, Q40.8, Q40.9, Q41, Q42, Q43.1, Q43.3-Q43.7, Q43.9, Q44, Q45, Q50.0, Q51, Q52.0-Q52.2, Q52.4, Q54.0-Q54.3, Q54.8, Q54.9, Q55.0, Q55.5, Q56, Q60.1, Q60.2, Q60.4-Q60.6, Q61, Q62.0-Q62.6, Q62.8, Q63.0-Q63.2, Q63.8, Q63.9, Q64, Q65.0-Q65.2, Q65.8, Q65.9, Q67.5, Q68.2, Q68.3-Q68.5, Q71-Q73, Q74, Q75.0, Q75.1, Q75.3-Q75.9, Q76.1-Q76.4, Q77, Q78, Q79.0, Q79.2-Q79.5, Q79.6, Q79.8, Q82.0-Q82.4, Q82.9, Q86.2, Q85, Q86.0, Q86.1, Q86.8, Q87.8, Q89.1, Q89.2, Q89.3, Q89.7-Q89.9, Q90-Q93, Q95.2, Q95.3, Q97, Q99 |
|  | Complex chronic conditions | B20-B23, D55, D561, D562, D570-D572, D58, D80-D84, D898, D899, E343, E70-E730, E74, E76-E79, E803-E807, E83-E85, E881, E882, E888, E889, F70, F72, F73, F842, G10-G12, G20, G23, G240-G242, G248, G250-G256, G318, G319, G40, G41, G71, G72, G80-G82, G901, G903, G904, G91, G940-G942, G95, G99, I42, I44, I45, I47-I49, I515, K44, K50-K51, K73-K74, K754, K758-K760, M41, N18, P27, P90 |
|  | Neonatal abstinence syndrome | P961 |
|  | Noxious influences | P04 |
|  | Perinatal infection | P35-P39 |
|  | Meningitis or encephalitis | G00-G09 |
|  | Necrotising enterocolitis* | P77 |
|  | Intraventricular hemorrhage* | P52, P912 |
|  | Retinopathy of prematurity* | H351 |
|  | Respiratory distress syndrome* | P22 |

*Only included in this group if infants were born <37 weeks gestation.

**Appendix Table 2: Risk factors for ED visits not resulting in an admission for infants in England and Ontario, 2010-2013**

|  |  | **Ontario (n=253,930)** | | **England (n=1,361,696)** | |
| --- | --- | --- | --- | --- | --- |
|  |  | **OR (95% CI)** | **p-value** | **OR (95% CI)** | **p-value** |
| **Gestational age at birth (weeks)** | Full term (39+) | 1 | <0.001 | 1 | <0.001 |
|  | Early term (37-38) | 1.12 (1.10-1.14) |  | 1.11 (1.10-1.13) |  |
|  | Late preterm (34-36) | 1.18 (1.13-1.23) |  | 1.12 (1.10-1.15) |  |
| **Newborn length of stay (days)** | <2 | 1 | <0.001 | 1 | <0.001 |
|  | 2-6 | 1.09 (1.07-1.12) |  | 1.10 (1.09-1.11) |  |
|  | 7+ | 1.18 (1.11-1.25) |  | 1.20 (1.17-1.23) |  |
| **Size for gestation** | Small (<10^th^ percentile) | 0.99 (0.96-1.01) | <0.001 | 0.99 (0.97-1.00) | 0.090 |
|  | Normal | 1 |  | 1 |  |
|  | Large (>90^th^ percentile) | 1.05 (1.02-1.08) |  | 1.00 (0.98-1.01) |  |
| **Maternal age (years)** | <=19 | 2.00 (1.91-2.10) | <0.001 | 1.63 (1.61-1.66) | <0.001 |
|  | 20-24 | 1.40 (1.36-1.44) |  | 1.29 (1.28-1.31) |  |
|  | 25-29 | 1 |  | 1 |  |
|  | 30-34 | 0.82 (0.81-0.84) |  | 0.84 (0.84-0.85) |  |
|  | 35-39 | 0.77 (0.75-0.79) |  | 0.76 (0.75-0.77) |  |
|  | >=40 | 0.73 (0.70-0.76) |  | 0.73 (0.72-0.75) |  |
| **Female sex** |  | 0.86 (0.85-0.87) | <0.001 | 0.86 (0.86-0.87) | <0.001 |
| **Primiparous mother** | | 1.01 (1.00-1.03) | <0.001 | 1.14 (1.13-1.15) | <0.001 |
| **Multiple birth** |  | 0.75 (0.70-0.80) | <0.001 | 0.70 (0.67-0.72) | <0.001 |
| **Deprivation quintile** | Most deprived | 1.24 (1.20-1.27) | <0.001 | 1.27 (1.25-1.29) | <0.001 |
|  | 2 | 1.16 (1.12-1.19) |  | 1.18 (1.17-1.20) |  |
|  | 3 | 1.12 (1.09-1.15) |  | 1.12 (1.11-1.14) |  |
|  | 4 | 1.07 (1.04-1.1) |  | 1.06 (1.05-1.07) |  |
|  | Most affluent | 1 |  | 1 |  |
| **Caesarean section** | | 1.02 (1.00-1.04) | 0.038 | 1.04 (1.02-1.05) | <0.001 |
| **Admission to Neonatal Intensive Care** | | 1.07 (1.04-1.11) | <0.001 | 1.03 (1.02-1.05) | <0.001 |
| **Season of discharge** | Jan-Mar | 1 | <0.001 | 1 | <0.001 |
|  | Apr-Jun | 0.99 (0.97-1.02) |  | 1.09 (1.08-1.10) |  |
|  | Jul-Sep | 1.02 (0.99-1.04) |  | 1.05 (1.04-1.06) |  |
|  | Oct-Dec | 1.00 (0.98-1.03) |  | 1.02 (1.00-1.03) |  |
| **Quarter-year of discharge** | | 1.00 (1.00-1.01) | 0.008 | 1.02 (1.02-1.02) | <0.001 |
| **Perinatal infection** | | 1.08 (0.98-1.19) | 0.125 | 1.02 (0.99-1.05) | 0.206 |
| **Prematurity related risk factor** | | 1.00 (0.92-1.09) | 0.988 | 1.04 (0.99-1.09) | 0.101 |
| **Neonatal medical condition** | | 1.19 (1.12-1.25) | <0.001 | 1.14 (1.11-1.17) | <0.001 |
| **Pregnancy risk factor** | | 1.10 (1.07-1.13) | <0.001 | 1.05 (1.04-1.07) | <0.001 |
| **Delivery risk factor** | | 0.99 (0.97-1.01) | 0.419 | 1.03 (1.01-1.04) | <0.0001 |
